# High throughput single cell analysis of mitochondrial heteroplasmy in mitochondrial diseases

**DOI:** 10.1038/s41598-020-67686-z

**Published:** 2020-07-02

**Authors:** Ryotaro Maeda, Daisuke Kami, Hideki Maeda, Akira Shikuma, Satoshi Gojo

**Affiliations:** 1 Department of Cardiovascular Medicine, Graduate School of Medical Science, Kyoto Prefectural University of Medicine, 465, Kajii cho, Kamigyo ku, Kyoto, 802-8566 Japan; 2 Department of Regenerative Medicine, Graduate School of Medical Science, Kyoto Prefectural University of Medicine, 465, Kajii cho, Kamigyo ku, Kyoto, 802-8566 Japan

**Keywords:** Biological techniques, Biotechnology, Genetics

## Abstract

Mitochondrial heteroplasmy, which fundamentally means intracellular heterogeneity of mitochondrial DNA (mtDNA), has been measured in a group of cells, regardless of intercellular heterogeneity. Ordinal methods for mitochondrial heteroplasmy cannot discriminate between an intercellular homogenic population composed of cells with similar intracellular heterogeneity for mtDNA and an intercellular heterogenic population composed of cells with different rates of mutated mtDNA. A high-throughput method to determine mitochondrial heteroplasmy in a single cell was developed by using droplet digital PCR with TaqMan polymerase in this study. This technique revealed that there are three different cell populations of cultured fibroblasts derived from patients with mitochondrial disease carrying a mutation in the mtDNA; cells with homoplasmy of either mutated or healthy mtDNA; and cells mixed with mutated and healthy mtDNA. The presence of intercellular heterogeneity, even in uniformed cultured fibroblasts, suggests that heterogeneity should exist among different kinds of cells. The diagnosis of intercellular heterogeneity with respect to mitochondrial heteroplasmy by this methodology could provide novel insight into developing a treatment strategy for mitochondrial diseases.

## Introduction

Mitochondrial diseases are a group of genetic heterogenous disorders characterized by dysfunctional mitochondria and currently cannot be cured; only palliative treatment to relieve symptoms are provided to patients^1^. Since the symbiosis of the progenitor of mitochondria and eukaryotes, α-proteobacteria and archaea, respectively, an array of genes in mitochondria were transferred into the nuclear genome that simultaneously evolved to acquire a 200,000-fold expansion in the number of genes^2^. Either the mutations in the nuclear gene constituting mitochondria or in the mitochondrial DNA (mtDNA) manifest mitochondrial diseases, which can affect any organ, develop at any age, and exhibit different degrees of severity^3^. Phenotypic variability in the case of a pathological mutation in mtDNA could be attributed to multiple copies of mtDNA that an individual cell possesses, ranging from 100,000 copies in an unfertilized oocyte to ~ 100 copies in sperm^4^. Although a small ratio of the mitochondrial genome with pathological mutations is pervasive, there seems to be a threshold, typically 60–80%, to manifest biochemical malfunctions^4^. The mixture of mutated and wild-type genomes is called heteroplasmy^5^. The severity of the diseases correlates with the level of heteroplasmy in the case of protein-coding gene mutations; an example of this is neurogenic muscle weakness, ataxia and retinitis pigmentosa (NARP), which is caused by the mutation of MT-ATP6. Heteroplasmy less than 70% does not exhibit a clinical phenotype, and heteroplasmy ranging from 70 to 90% manifests symptomatic NARP that might remain stable into adult life; extreme heteroplasmy of more than 90% develops as Leigh syndrome^6^. On the other hand, mtDNA mutations in tRNA genes manifest with high clinical variability, which cannot be explained by heteroplasmy^7^.

Given that the mixing rate of healthy and mutant genotypes in a single cell is constant in all cells, the assay targeting a population accurately portrays the situation of each cell. However, it was reported that the mixing rates are not constant, and the mutations are even various among neurons^8^. Recently, intracellular and intercellular mtDNA heteroplasmy, which are termed microheteroplasmy and macroheteroplasmy, respectively, have been clearly defined^9^.

There are many methods to detect genotype DNA sequence variances, including single nucleotide polymorphisms (SNPs), which have been utilized to diagnose cancer^10^, genetic disorders^11^, and infectious diseases^12^. Amplification refractory mutation system (ARMS) is based on oligonucleotides that have a mismatched 3′ residue to the template that does not efficiently extend the PCR strand^13^. ARMS needs only the primer to be designed with either a 3′-matched or -mismatched end and does not require isotopes, restriction enzymes, sequence reactions, or specialized instruments. The specificity for ARMS primers depends upon the sequence of the template^14^; therefore, some modification with additional mismatches upstream to the 3′ end improved the specificity without reducing the simplicity. Quenching, which is defined as a decrease in the fluorescence intensity of a given fluorophore, is utilized for signal detection in various PCR-based SNP assays^15^. TaqMan quantitative PCR was applied to detect DNA sequence variance in combination with a sequence-specific probe^16^. The oligonucleotide probe, which possesses both a fluorophore and quencher at the opposite end and exhibits little fluorescence in the aqueous phase, is digested by the activity of the 5′ exonuclease of the TaqMan Polymerase in the extension process of PCR, resulting in emitting fluorescence from the dissociated fluorophore. Multiplex detection of several kinds of mutations is feasible in a single reaction tube by using different fluorophores^17^. To detect a quite rare variant, an array of modifications has been applied to the combination method with primers and probes, and these include CataCleave^18^, Scorpion-ARMS^19^, and PNA-LNA PCR clamp^20^, which have a sensitivity of 5%, 1%, and 1%, respectively. A double-strand DNA fragment has an inherent melting temperature, which is applied to a probe-based fluorescence melting curve analysis. Using a probe with a fluorophore and a quencher at different ends, the difference in the differentiating signal with the temperature of a probe-template hybrid are plotted in relation to temperature, resulting in a discriminated peak that is dependent on the sequence even in a single nucleotide^21^.

Third-generation PCR, a digital PCR based on microwell chip and microfluidic techniques, emerged to detect one target sequence with an analytic system based on the Poisson distribution^22^. The droplet digital PCR (ddPCR) using the water-in-oil droplet technique exhibited a higher sensitivity to distinguish rare mutations with a 0.001% sensitivity^23^. It has also been utilized to measure the mitochondrial DNA copy number using eluted nucleotides^24^ or lysed cells^25^. Although these assays claimed to provide single cell resolution by harnessing the limiting dilution, the results are based upon the hypothesis that all cells possess the same heterogeneity of mtDNA. Although the abovementioned methodologies have improved the specificity and sensitivity, the results might not accurately express the situation in each cell but rather be an integration of those in each cell in the population.

Single-cell biology has emerged so rapidly in various fields and has contributed to providing deeper insights into life science. Single-cell ddPCR (sc-ddPCR), which takes advantage of an intact cell instead of lysed cells or eluted nucleotides and is executed in a single droplet enclosing a single cell, has been developed by using a droplet-to-digital device. To examine a deletion in mtDNA, this technology was applied to a single skeletal muscle cell collected by laser-capture microdissection (LCM) from muscle biopsies^26^. In ex vivo gene therapy using hematopoietic stem cells, sc-ddPCR enabled the tracing of gene-modified donor cells^27^. In bacteriology, multiplexed single-cell ddPCR was applied to detect different alleles^28^. On the other hand, next-generation sequencing technology opened a new field of single-cell biology, and even within a single mitochondrion, it revealed heteroplasmy in mtDNA^29^. In this study, we introduced high-throughput sc-ddPCR as a detection method for a heteroplasmy within a single cell. This analysis reveals how the mutated mtDNA is truly distributed in each cell and the genotype with respect to mtDNA in a single cell, not in a population.

## Results

### Single nucleotide polymorphism assay in whole cells

**Figure 1:**
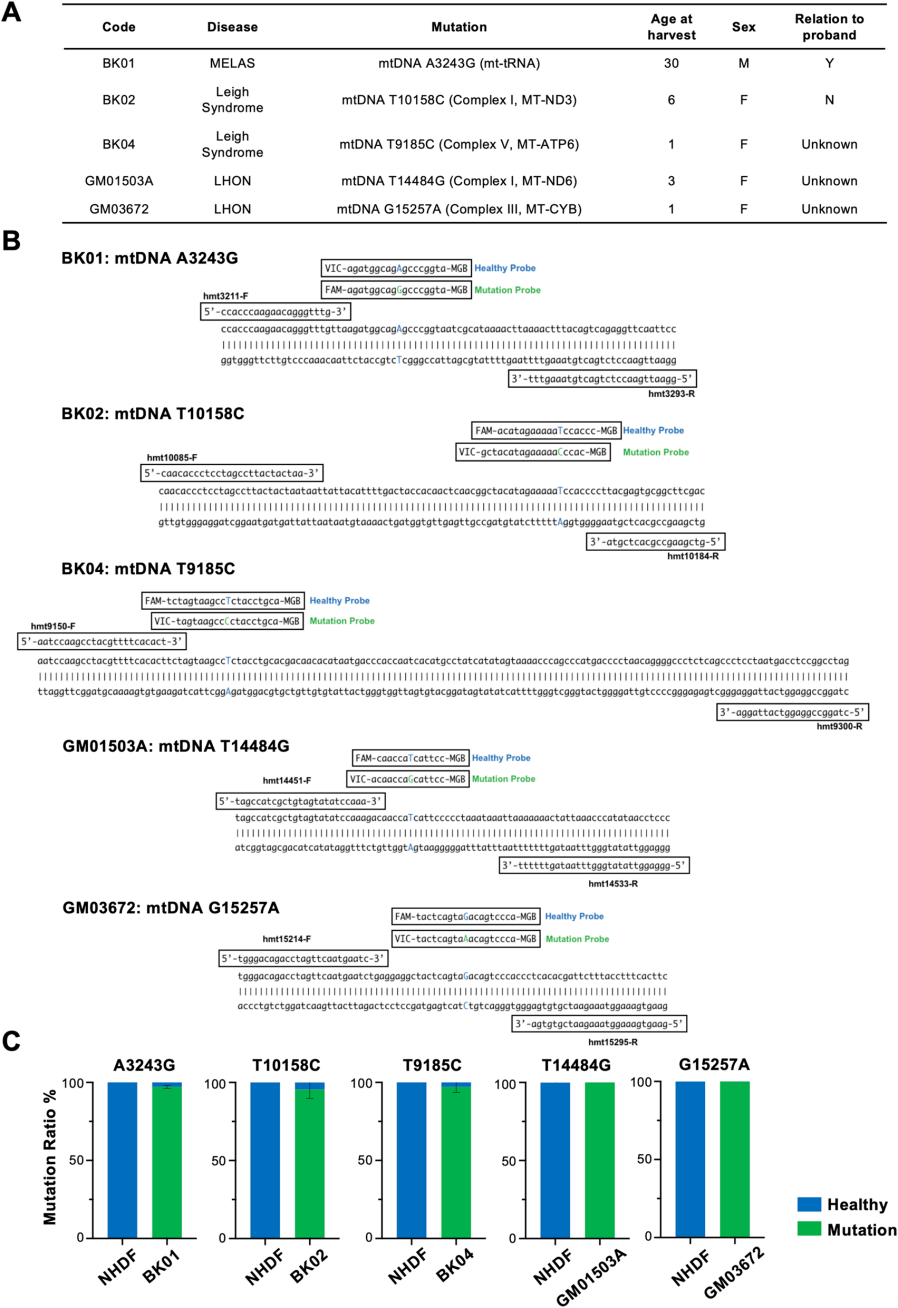
(**A**) Clinical and genetic characteristics of patients from whom primary human dermal fibroblasts have been derived. (**B**) The set of primers and probes is shown on the amplified fragments in the TaqMan SNP Genotyping Assay. (**C**) Heteroplasmy rates detected by TaqMan SNP Genotyping Assay in each fibroblast.

We utilized 5 primary fibroblasts derived from patients with mitochondrial diseases, which are caused by a mutation in mtDNA (Fig. 1A). The TaqMan single nucleotide polymorphism (SNP) assay was chosen to determine the heteroplasmy in mtDNA of target cells with respect to simplicity because the same assay is easily adopted for ddPCR. A set of primers was designed to target the SNP-encompassing region and to amplify 83 bp, 100 bp, 151 bp, 83 bp, and 82 bp sequences for BK01, BK02, BK04, GM01503A, and GM03672, respectively. To discriminate and quantify healthy genotypes compatible with the Cambridge Reference Sequence (CRS) and mutated mtDNA separately, two TaqMan probes with either a FAM or VIC fluorescent dye at the 5′ end and a nonfluorescent quencher at the 3′ end were designated in combination with the minor groove binders (MGBs) to maximize the difference in the melting temperature in each kind of fibroblast. The amplified sequences were subcloned into a plasmid either to depict the standard line for quantification of the target sequence in the conventional TaqMan SNP genotyping assay or to draw threshold lines in the sc-ddPCR assay (Fig. 1B). The mean heteroplasmy from five fibroblasts was 97.1%, 95.5%, and 97.1% for BK01, BK02, and BK04 (N = 6), respectively, and 100% for both GM01503 and GM03672 (N = 3) (Fig. 1C, Supplemental Fig. S1). The specificity of each primer–probe set was examined by using NHDFs that had been verified to possess healthy sequences in the target position by NGS. The heteroplasmy rate in NHDFs was negligible for all mutant probes.

### sc-ddPCR single cell assay

**Figure 2:**
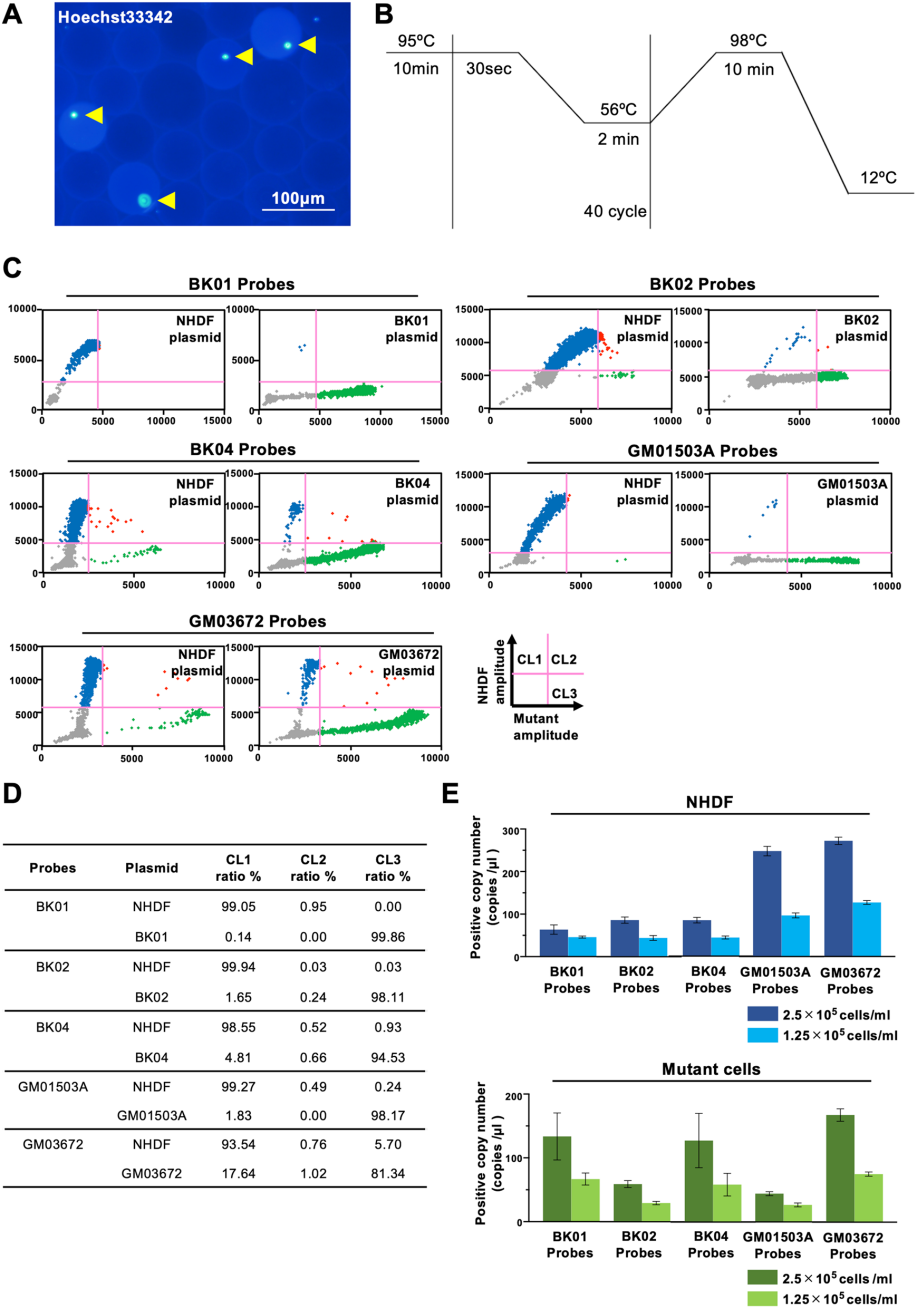
(**A**) Representative image of droplets containing a single cell. Arrowheads show nuclei of cells dyed by Hoechst 33342. (**B**) sc-ddPCR protocol that we used for this work. (**C**) Threshold line setting for the set of probes. Threshold lines were set by ddPCR using plasmids carrying a NHDF-specific (healthy) target sequence (NHDF plasmid) and mutation-specific target sequence (BK01, BK02, BK04, GM01503A, and GM03672 plasmids). (**D**) Results of ddPCR using plasmids for threshold line setting. (**E**) Threshold lines validated by the proportional relations between the cell copy number of the positive signals above the threshold and the loaded cell number. The upper figure shows the cell copy number of NHDF-specific (healthy) target sequences detected by each set of probes when encapsulating NHDFs at a concentration of 2.5 × 10^5^/ml or 1.25 × 10^5^/ml. The lower figure shows the cell copy number of the mutant-specific target sequence detected when encapsulating mutant cells at a concentration of 2.5 × 10^5^/ml or 1.25 × 10^5^/ml.

Droplets were generated by using various concentrations of the cell suspension so that they included either a single cell or no cells. The concentration of 1 × 10^5^ cells per milliliter was optimal (Fig. 2A). Normal human dermal fibroblasts (NHDFs), which were sequenced for mtDNA and ensured to have a compatible sequence to CRS in MT-ND3, MT-ATP6, tRNA for leucin, MT-ND6, and MT-CYB, were utilized as the control. All five kinds of primer and probe sets for mutations in m3243, m10158, m9185, m14484 and m15257 were designed based on the predicted melting temperature between the probe and template. The optimized PCR protocol was designed to minimize the nonspecific binding of probes and maximize the fluorescence intensity (Fig. 2B). To analyze two fluorescence data in a 2-dimensional plot, we designated how to set a quadrant line for each target sequence by using a plasmid carrying it (Fig. 2C). This plot provides specificity for a probe that is not for a target template, so the specificities of the probe for BK01, BK02, BK04, GM01503, and GM03672 in combination with the probe for NHDFs are summarized in Fig. 2D. The threshold lines were validated by the proportional relation between the cell number of the positive signal and the loaded cell number (Fig. 2E).

**Figure 3:**
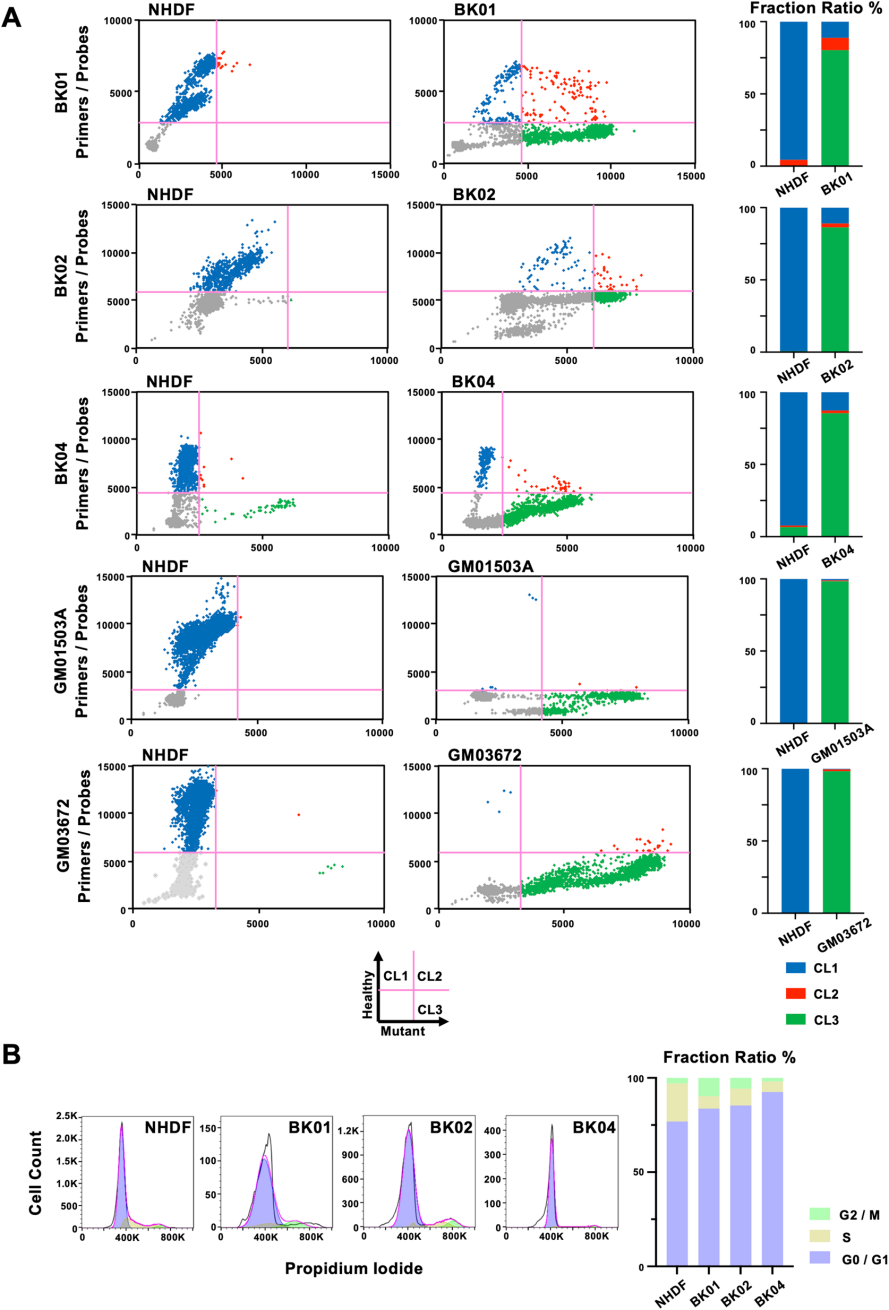
(**A**) Quadrant analysis of mtDNA heteroplasmy in a single cell and the bar graph expressing the ratio of each quadrant for each primary fibroblast from patients. (**B**) FACS analysis of the cell cycle in three primary fibroblasts from patients, using NHDFs as a control.

We depicted the results in a quadrant format with a healthy signal as the y-axis and a mutant signal as the x-axis. The quadrant analysis shows cells possessing only healthy mtDNA in the upper left (cluster 1: CL1), cells possessing both mutant and healthy mtDNA in the upper right quadrant (cluster 2: CL2), and cells possessing only mutant mtDNA in the lower right quadrant (cluster 3: CL3). The lower left quadrant exhibits droplets without a cell (Fig. 3A, Supplemental Fig. S2). All SNP assays using sc-ddPCR were performed in triplicate. The quadrant analysis in BK01 showed that a major part of cells was homoplasmy in the mutant mtDNA plotted in CL3, but a minor part of cells had two kinds of mtDNA, the mutant and healthy mtDNA, plotted in CL2, which is the state of a microheteroplasmy (Fig. 3A, upper panel). Moreover, a population of cells constituted solely of healthy mtDNA existed in cells were considered to be a homogeneous population. BK02 and BK04 contained the cell population with both the mutant and healthy mtDNA plotted in CL2 at a rate of 2.73% and 1.88%, respectively (Fig. 3A, middle panel). Both GM0503A and GM03672 were homoplasmic of mutant mtDNA (CL3) at approximately 98.3%, whereas the remnants exhibited either heteroplasmy or homoplasmy of healthy mtDNA (Fig. 3A, lower panel).

### Cell cycle analysis

In the quadrant analysis, there were two distinct fractions in CL1 and CL3, although it was difficult to judge whether two populations were in CL2 due to small events. Cell cycle analysis revealed that the fractions of S phase in the three cell lines were less than half that found in NHDFs. The sum of the G2/M and S phases ranged from 10 to 20% in diseased fibroblasts (Fig. 3B). The ratios of the two fractions were nearly even, suggesting that the duplicated content of mtDNA might occupy half of the cell cycle.

## Discussion

This study provides a high-throughput method to evaluate the heteroplasmy of mtDNA in a single cell, not quantitatively, but by the presence or absence of the mutated mtDNA. This sc-ddPCR could reveal the existence of mtDNA variances as low as 1% frequency in a single cell without cloning steps. The time to complete sc-ddCPR and analyze its data should be less than a few hours, the running cost for each sample is tens of dollars, and the procedure in sc-ddPCR is similar to conventional qPCR. Because we harnessed the recombinant plasmid carrying a target sequence to draw the standard line in the conventional TaqMan SNP genotyping assay, it was diverted in sc-ddPCR. In the case of independent sc-ddCPR, the target sequence for the threshold line should use chemically produced oligonucleotides with respect to cost, labor, and time. The existence of the minor population of homoplasmy with healthy mtDNA suggests that the patient’s skin might hold fibroblasts with a healthy homogeneous haplotype of mtDNAs. Moreover, the minority of intracellular heteroplasmy in all fibroblasts could be suggestive of the development and progression of mitochondrial diseases in a single cell with a life that has unsolved questions. The heteroplasmy in either dividing cells or nondividing cells changes with time via various mechanisms, which affect the development and progression of the disease. In the context of cancer, treatment with anti-cancer drugs targeted to specific molecules along with companion diagnostic immunoassays have been shown to assist in determining whether different individual treatment regimens are indicated. In the treatment of mitochondrial diseases, development of similar companion diagnostics is still required^30^. Although recent advances in biomarkers for mitochondrial diseases have established the effectiveness of fibroblast growth factor 21 (FGF21)^31^ and growth/differentiation factor 15 (GDF15)^32^, the regain of wild-type mtDNA and the removal of mutation burden in affected cells should be an optimal indicator of a successful cure. Heteroplasmy should be useful not only for a diagnosis of mitochondrial diseases but also for an estimation of the effectiveness in the treatment process.

Methods to detect heteroplasmic variations in the mitochondrial genome are divided into two modalities: next-generation sequencing (NGS) for unrecorded mutations and PCR-based detection for recorded mutations. A major portion of genomic samples originate from the nucleus (more than 99% of the total DNA), and this could disturb outcomes in both methods to detect the diversity of the mitochondrial genome. Cesium chloride density centrifugation was initially utilized for the enrichment of the mitochondrial genome^33^. Mitochondrial isolation with centrifugation^34^ was examined for the purpose of mitochondrial genome enrichment, which has not completely prevented contamination of the nuclear genome. The whole mitochondrial genome has been mapped by either long-range PCR^35^ or overlapping fragment amplification^36^, both of which hold clonal amplification of errors introduced by polymerase^37^. Another mitochondrial genome enrichment protocol was introduced for heteroplasmy research to utilize DNA polymerase of bacteriophage phi29, which possesses strand displacement activity, proof-reading activity and generation of very long synthesis products, resulting in more efficient amplification of circular DNA than linear DNA^38^. MitoRS enriched the mitochondrial genome by using phi29, and the sequence revealed single nucleotide variants at a 1% frequency^39^. Although methyl-specific endonucleases to delete the nuclear genome that is more methylated than the mitochondrial genome were utilized for mitochondrial genome enrichment^40^, a significant amount of the nuclear genome could remain, and mitochondrial genomes could lose. Another enzymatic digestion specific to the linear nuclear genome, exonuclease V, was applied to enrich the mitochondrial genome, which offered the ideal library introducing fragments of only the mitochondrial genome with barcoded adapters to NGS^41^. The abovementioned procedures are time-consuming, require a large quantity of samples, and are limited in use, especially being unadaptable to single-cell research.

Although NGS is an essential tool to detect both de novo and recorded mutations for rare genetic diseases, including mitochondrial diseases^42^, the presence of nuclear mtDNA homologous sequences (NUMTs) could hinder the accuracy due to false heteroplasmic variations from NUMTs^43^. NUMTs have been annotated in the human reference genome^44^ but depend upon an individual sample, and the choice of the reference crucially impacts the outcomes in detecting heteroplasmic variances^45^. To examine low-level heteroplasmic variations, how to align the raw reads of the whole genome has been devised by a two-step alignment to eliminate the effects of NUMTs, reaching the power of detecting heteroplasmy as low as 0.2% in two cell lines^46^. Polymorphism ratio sequencing that utilizes Sanger sequencing technology could not identify heteroplasmic variance less than 5%^47^. Duplex sequencing technology that reduces errors by independently tagging and sequencing each of the two strands of DNA achieved a detection limit of 0.1%^48^. Single-cell sequencing that was applied to determine mtDNA mutations in hematopoietic cells was executed following single-cell sorting with flow cytometry and direct two-step nested PCR amplification without cloning but was unable to recognize signals less than 10%^49^. Single-cell cloning and sequencing, where eluted and amplified DNA fragments are cloned into plasmids and transformed into *E. coli*, and the positive clones are sequenced, was applied to single neurons and glial cells^50^. However, the high biased mutation frequency in replication could not provide the accuracy to reveal rare variances. Currently, single-cell sequencing remains to be solved for low levels of heteroplasmy variances, and even in conventional NGS, it is too expensive to generate data for laboratory experiments, such as acquiring them over a time course. On the other hand, although PCR-based methods can be generally applied and only record sequence variances, their simplicity, swiftness, and inexpensiveness allow them to be utilized as a useful tool. Amplification refractory mutation system quantitative PCR that amplifies templates with two allele-specific upstream primers and one downstream primer demonstrated the same detection limit of 0.1% for duplex sequencing^51^. Digital PCR conceptionally emerged from single-molecule PCR, which can discover de novo mutations but cannot recognize variances less than 7%^52^, and evolved to droplet digital PCR by combining microfluidics devices^53^. Enrichment of amplified products on magnetic beads in emulsions, called beads, emulsions, amplification and magnetics systems, improved the detection limit to 0.01%, which are compatible with ddPCR^54^. Although sc-ddPCR with dual fluorescence colors for heteroplasmy variances is inferior to ddPCR with respect to sensitivity, ddPCR that uses eluted genetic materials from pooled cells, which other current sophisticated PCR-based methods for detecting heteroplasmy, such as CataCleave, Scorpion-ARMS and PNA-LNA PCR clamp, also utilize as initial material, merely provides average values in a group and might mislead researchers regarding the effects of mtDNA variances. This sc-ddPCR is a first modality to execute a high-throughput detection system for intercellular heterogeneity, although the extent of intracellular heteroplasmy is not quantified. In this study, the reduced population of cells with heteroplasmy compared with those with either healthy or mutated mtDNA suggests that heteroplasmy status might be unstable, although more patients and other kinds of cells should be examined to confirm this possibility. The quantitative measurement of cells with healthy or mutant homoplasmy, or heteroplasmy, could provide cues regarding how mitochondrial DNA replication and segregation are regulated. For perspective, the next generation of ddPCR technology provided by BioRad will soon be released, being multiplex with multi-laser excitation and multi-emission detection. In addition to detecting multiple mutations, the new machine enables the simultaneous detection of phenotypes and genotypes in a single cell, such as mitochondrial ROS generation, by using dye and mitochondrial heteroplasmy, which may help to characterize mitochondria in a single cell and help to elucidate not only mitochondrial diseases but also aging processes and tumorigenesis related to mtDNA mutations.

## Materials and methods

### Cell culture

Normal human dermal fibroblasts (NHDFs) were obtained from Lonza (Walkersville, MD, USA). Mitochondrial disease patient-derived skin fibroblasts (BK01/02/04) were kindly provided by KOINOBORI Associate Inc., which supports the research for mitochondrial diseases under the approval from the ethical committees of both our institution and KOINOBORI. In addition, two additional fibroblasts (GM01503A and GM03672) were obtained from Coriell Institute for Medical Research (Camden, NJ, USA). The clinical characteristics of these primary cells are summarized in Fig. 1A. NHDFs were maintained in Dulbecco’s modified Eagle’s medium (Thermo Fisher Scientific, Waltham, MA, USA) supplemented with 10% fetal bovine serum (Thermo Fisher Scientific) and 1% penicillin/streptomycin. BK01 cells were cultured in Fibroblast Basal Medium (FBM) supplemented with FGM-2 SingleQuots (hFGF-B, insulin, FBS and gentamicin/amphotericin-B) (Lonza, Walkersville, MD, USA). BK02 and BK04 were cultured in Dulbecco’s modified Eagle’s medium low glucose (Thermo Fisher Scientific) supplemented with 10% fetal bovine serum (Thermo Fisher Scientific) and 1% penicillin/streptomycin. GM01503A and GM03672 were cultured in Minimum Essential Medium (Thermo Fisher Scientific) supplemented with 15% fetal bovine serum (Thermo Fisher Scientific), 1% Non-essential Amino Acid Solution (Thermo Fisher Scientific) and 1% penicillin/streptomycin. All cells were incubated at 37 °C in a humidified 5% CO_2_ incubator.

### Cell cycle analysis by flow cytometry

Cells were trypsinized, suspended in culture medium, centrifuged (1,000 rpm, 5 min) to pellet, and resuspended in PBS. The resuspended cells were added into 4% paraformaldehyde (FUJIFILM Wako Pure Chemical Corporation, Osaka, Japan) and fixed for at least 15 min at room temperature. After fixation, the cells were centrifuged (1,500 rpm, 5 min), resuspended in propidium iodide solution composed of 50 μg/ml propidium iodide, 0.1 mg/ml RNase A, 0.05% Triton X-100 and PBS, and incubated for 40 min at 37 °C. After washing with PBS, the cells were pelleted (1,500 rpm, 5 min), and resuspended with PBS. The samples were immediately analyzed by flow cytometry. Cell cycle phase distribution was determined using FlowJo software (Becton, Dickinson and Company, Franklin Lakes, NJ, USA).

### Heteroplasmy analysis for mitochondrial DNA mutation

Heteroplasmy of mitochondrial DNA was determined by TaqMan SNP Genotyping Assay^16^. Wild-type and mutant allele-specific TaqMan probes and primers were designed and produced by Thermo Fisher Scientific. The two probes were labeled with different fluorophores (FAM and VIC) with a quencher attached at the other end. Genomic DNA was extracted from cells by using NucleoSpin Tissue (Takara Bio Inc., Shiga, Japan). The extracted genomic DNA (100 ng) was used for quantitative PCR by mixing with the forward and reverse primers, the probes, and the TaqMan Genotyping Master Mix (Thermo Fisher Scientific) on a CFX connect real-time system (Bio-Rad Laboratories, Inc., Hercules, CA, USA) under the following conditions: 40 cycles of PCR (95 °C for 15 s and 60 °C for 1 min) after the initial denaturation (95 °C for 10 min). A calibration curve was created by the abovementioned quantitative PCR using the plasmid of decided copy numbers containing the amplified targeted mtDNA fragments for either wild-type or mutant sequences. The primers used in this experiment are listed in Fig. 1B.

### Single cell droplet digital PCR (sc-ddPCR)

The sc-ddPCR system commenced with the encapsulation of a single cell into one oil droplet and then proceeded to the PCR step with a set of primers and fluorescent probes, which are the same as those used in the TaqMan SNP genotyping assay, using TaqMan Polymerase with the 5′ to 3′ exonuclease, which releases the fluorophore from the probe, followed by the detection of the fluorescent signal in the droplets. The PCR mixture consisted of 4 μl resuspended cells at a concentration of 2.5 × 10^5^/ml or 1.25 × 10^5^/ml, 10 μl 2 × ddPCR Supermix (Bio-Rad), wild-type and mutant allele-specific TaqMan probes at a concentration of 0.25 μM, primer mixtures at a concentration of 0.9 μM for the target gene, and nuclease-free water for a final volume of 20 μl. Droplets were generated using the Bio-Rad QX200 system (Bio-Rad) following the manufacturer’s instructions. The reactions were transferred to a 96-well plate (Eppendorf Corp., Hamburg, Germany) for the PCRs using a Thermal Cycler (Bio-Rad) under the following conditions: amplification was carried out at a regular ramp rate of 2.0 °C /s at 95 °C for 10 min followed by 40 cycles of 30 s at 95 °C plus 2 min at 56 °C. The final enzyme deactivation step occurred at 98 °C for 10 min. The 96-well plate was transferred to a QX200 Droplet Reader (Bio-Rad), and the number of fluorescent-positive droplets were analyzed. Each droplet was analyzed individually using a two-color detection system (set to detect FAM and VIC). The fluorescent droplets were counted to provide an absolute quantification of target mtDNA in digital form using QuantaSoft software 1.7 (Bio-Rad). We added various numbers of targeted cells to the PCR mix and generated droplets to ensure single-cell encapsulation. A total of 500 cells per sample were successfully encapsulated into droplets, and single-cell encapsulation was observed. The threshold line for each fluorescence was determined by separate experiments, where droplets enclosed a plasmid carrying a target sequence (5,000 copies/a single sample, in which the majority of the droplets were null while the minority of droplets contained a single copy), instead of a single cell, and both healthy and mutant probes with different fluorescences were executed for the same ddPCR protocol as the designated sc-ddPCR protocol. When the results are plotted in 2 dimensions, a quadrant line is designated for the single positive cluster in the corresponding target-probe combination to provide positive signals of no more than 1% in the alternative fluorescence at the next quadrant. By drawing the threshold lines, the specificity for the probe is given as follows: 1-(positive fraction in an alternative fluorescence/all events in droplets, excluding null droplets, in the corresponding target-probe combination).

### Patients characteristics

Five kinds of cells derived from patients diagnosed with a mitochondrial disease were examined in this study. The characteristics of these cells are summarized in Fig. 1A. These primary fibroblasts of the BK series were isolated from the skin biopsies of patients and were established as cultured cells after informed consent was obtained from all the patients or from a parent or legal guardian if the patient was under 18 years old and was based on the acceptance of the ethical committee from KOINOBORI Associate Inc., which is a nonprofit organization for mitochondrial diseases in Japan. These cells were provided for our research. This study was conducted in accordance with relevant guidelines and regulations, and was accepted by the institutional ethical committee in Kyoto Prefectural University of Medicine (#ERB-C-1010). Two other cell lines, GM01503 and GM03672, were purchased from Coriell Institute for Medical Research. BK01 was derived from a 30-year-old female patient with mitochondrial myopathy, encephalopathy, lactic acidosis, and stroke-like episodes (MELAS), whose culprit is the mutation of A to G in m3243 in the tRNA for leucine. Two other fibroblasts originated from female patients with Leigh syndrome who were 6 and 1 years of age. One patient (BK02) has the mutation of T to C in m10158 that is located in the mitochondrially encoded NADH dehydrogenase 3 (MT-ND3), which constitutes the respiratory chain complex I, also known as NADH dehydrogenase (ubiquinone), which consists of 37 nuclear and 7 mitochondrially encoded subunits. Another patient (BK04) has the mutation of T to C in m9185 that is located in the mitochondrially encoded ATP synthase membrane subunit 6, which encodes the ATP synthase F_0_ subunit 6 (MT-ATP6), the subunit of the F1F0 ATPase that is also known as complex V, consisting of 14 nuclear and 2 mitochondrial encoded subunits. The inheritance of the proband in BK01 was from her mother, and the mutation of BK02 was de novo. The inheritance of BK04 was not determined. GM01503 and GM03672 were derived from patients suffering from Leigh syndrome, which was confirmed with a clinical summary and case history. Because there was no sequence information for either cell line, the whole mitochondrial genome was sequenced and referenced against the MITOMAP database^55^. Variant m.14484 T > G in the MT-ND6 gene in GM01503, which results in the change of the methionine from position 64 to a valine, could be pathogenic because the change m.14484 T > C (p.Met64Val) has been described as one of the most frequent mutations associated with LHON. Variant m.15257G > A in the CYTB gene, which results in the change of aspartic acid from position 171 to asparagine, could be causative because the mutation was initially described as one of the primary mutations of LHON and associated with some mitochondrial diseases. Based on the sequencing data, both cell lines showed homoplasmy with mutated mtDNA at the abovementioned positions.

## Supplementary information

Supplementary file1 (DOCX 3962 kb)

## Data Availability

Raw data are available on request.
