# Supplementary material for: High throughput single cell analysis of mitochondrial heteroplasmy in mitochondrial diseases
Source: Sci Rep. 2020 Jul 2;10:10821. doi: 10.1038/s41598-020-67686-z (PMC7331593; doi:10.1038/s41598-020-67686-z)
Supplement: Supplementary file 1 — Supplementary file1 (DOCX 3962 kb) [file 41598_2020_67686_MOESM1_ESM.docx]

**High Throughput Single Cell Analysis of Mitochondrial Heteroplasmy**

**in Mitochondrial Diseases**

Ryotaro Maeda^1^, Daisuke Kami^2^, Hideki Maeda^1^, Akira Shikuma^1^,

Satoshi Gojo^2^

1: Department of Cardiovascular Medicine, 2: Department of Regenerative Medicine,

Graduate School of Medical Science, Kyoto Prefectural University of Medicine

**Supplemental Figures**


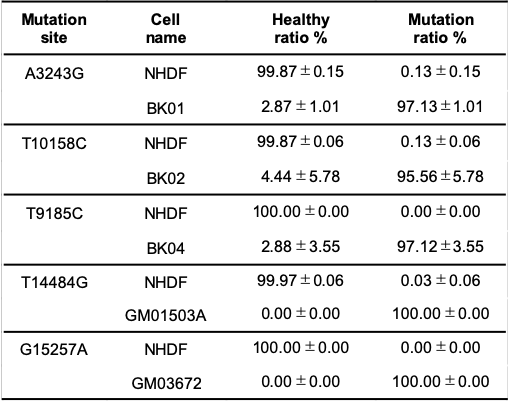


**Figure S1. Results of TaqMan SNP genotyping assay.**

Heteroplasmy rates of mtDNA detected by TaqMan SNP genotyping assay in each fibroblast are provided in Figure S1. Data are means ± SD of six different experiments for BK01, BK02 and BK04, and three different experiments for the other fibroblasts.


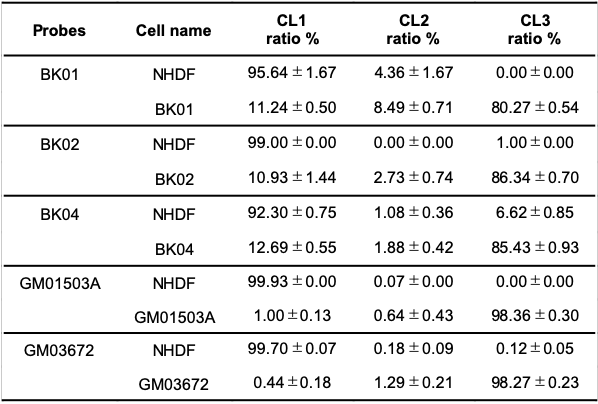


**Figure S2. Results of sc-ddPCR.**

Heteroplasmy rates of mtDNA in a single cell detected by sc-ddPCR in each fibroblast are shown in Figure S2. Data are means ± SD of triplicate samples.

**
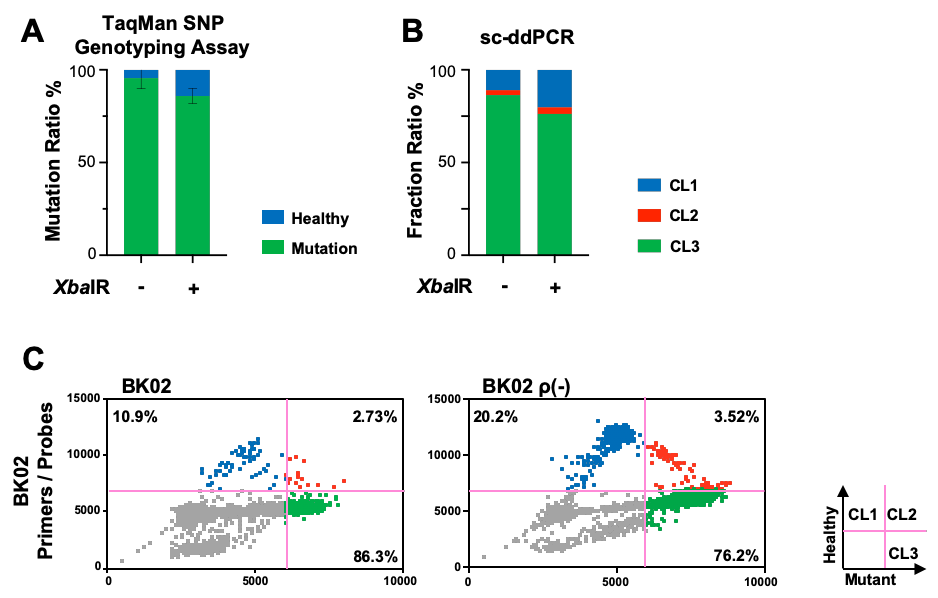
**

**Figure S3. Heteroplasmy shift of BK02 after *Xba*IR treatment.**

(A) Heteroplasmy rates detected by TaqMan SNP Genotyping Assay in BK02 and BK02 genetically modified by *Xba*IR fused with mitochondria transfer sequence (MTS) (BK02 ρ(-)). Data are shown as the means ± SD of six different experiments for BK02 and three different experiments for BK02 ρ(-). (B, C) Results of sc-ddPCR for BK02 and BK02 ρ(-). The bar graph expresses the average ratio of each quadrant in triplicate experiments (B). Representative images of the quadrant analyses are shown for mtDNA genotyping in a single cell (C).

In addition to 5 kinds of primary cultured cells that possess high heteroplasmy, we examined cells with moderate heteroplasmy to verify this technology. In a separate experiment, we recognized that unbiased significant reduction of mtDNA in cells with a heteroplasmy (called ρ(-) that means petit of mtDNA), which is achieved with gene transfer of endonuclease with MTS, could induce a heteroplasmic shifting (unpublished data). A strategy to achieve heteroplasmic shifting with endonucleases fused to MTS to date has targeted a mutation sequence to destroy the mutated mtDNA, resulting in reduced heteroplasmy ^1-3^. BK02 was genetically modified by an endonuclease, *Xba*IR, fused to MTS, which digests at 5 points that are not related to the mutation in BK02 based on the Cambridge Reference Sequence in human mtDNA, resulting in a significant reduction in mitochondrial copy number and a heteroplasmy shift from over 95% to approximately 85% that was estimated by the TaqMan SNP genotyping assay following 6 days with gene transfer. The resultant cells, ρ(-) cells, were analyzed by sc-ddPCR and revealed that homoplasmy in the healthy genotype drastically increased to approximately 20% and intracellular heteroplasmy with both genotypes emerged to approximately 3.5%. These results demonstrate that sc-ddPCR can adequately estimate moderate heteroplasmy.

References

1. Tanaka, D. M.; Borgeld, H.-J.; Zhang, J.; Muramatsu, S.-i.; Gong, J.-S.; Yoneda, M.; Maruyama, W.; Naoi, M.; Ibi, T.; Sahashi, K., Gene therapy for mitochondrial disease by delivering restriction endonucleaseSmaI into mitochondria. *Journal of biomedical science* **2002,** 9, (6), 534-541.

2. Bayona-Bafaluy, M. P.; Blits, B.; Battersby, B. J.; Shoubridge, E. A.; Moraes, C. T., Rapid directional shift of mitochondrial DNA heteroplasmy in animal tissues by a mitochondrially targeted restriction endonuclease. *Proc Natl Acad Sci U S A* **2005,** 102, (40), 14392-7.

3. Bacman, S. R.; Williams, S. L.; Garcia, S.; Moraes, C. T., Organ-specific shifts in mtDNA heteroplasmy following systemic delivery of a mitochondria-targeted restriction endonuclease. *Gene Ther* **2010,** 17, (6), 713-20.
